# Machine learning long-term electricity demand forecasting system for strategic energy investments

**DOI:** 10.1038/s41598-026-45123-x

**Published:** 2026-04-15

**Authors:** May Haggag, Karim Abdelhady, Minas Guirguis, Maram Saudy, Wael El Dakhakhni

**Affiliations:** 1 Construction Engineering Department, The American University in Cairo, Cairo, Egypt; 2 School of Computational Science & Engineering, McMaster University, Hamilton, Canada

**Keywords:** Electricity Consumption Forecasting, Energy Investment, Time Series Analysis, Data-Driven Energy Forecasting, Energy science and technology, Engineering, Environmental social sciences, Mathematics and computing

## Abstract

The Energy Institute’s 2025 report revealed that the global electricity consumption is rising by 4% annually, with regional variances driven by complex economic, social and climatic factors. Given the critical role of long-term consumption forecasting in informing energy investment decisions and ensuring the resilience of national power infrastructure against future demand fluctuation risks, the development of demand prediction systems that are both accurate and robust is imperative. Current univariate—assuming stable demand patterns, and multivariate models—constrained by narrow feature sets, possess a limited ability to capture cross-domain interactions among economic, social and climatic factors. To address these gaps, this study introduces a forecasting system that encompasses three-phases: *i*) Data Acquisition and Structuring; *ii*) Predictive Modeling and Optimization; and *iii*) Model Evaluation and Analysis. The developed system integrates multi-domain predictors, including electricity generation, as well as economic, social and climatic features, while also leveraging deep learning and SHAP-based interpretability to ensure accurate and robust predictions and provide insights into feature contributions. Deployed on Egypt using data from 2000 to 2023, the system results indicate that population and GDP per capita are the primary drivers of electricity demands, with generation capacity and external debt exerting secondary influences. The system model achieved a mean R²=0.83 across multiple random weight initializations and a root mean square error of 4.1% of the mean testing value, demonstrating its reliability for long-term scenario-based planning, strategic investment, and energy security policy formulation.

## Introduction

As urban populations grow and economies expand, the demand for reliable, affordable, and sustainable electricity continues to rise^1^. The Energy Institute’s 2025 report revealed that global electricity consumption is growing at 4% annually, with regional variation driven by variances in economic development, social statuses, climate conditions, and energy policies^2^. Empirical analyses further highlight that economic and population growth significantly influence electricity consumption as 1% increase in economic growth corresponds to a 0.49–0.81% rise in consumption, while 1% increase in population results in an increase of 1.23–1.59%^3^. Moreover, the exceptional consumption growth in 2024 was attributed to the observed hotter temperatures which resulted in an increase of 0.7% in global electricity demand^4^. Additionally, electricity demand is expected to increase by 3.4% annually between 2024 and 2026, outpacing projected global GDP growth of 3.1%, and long-term forecasts indicate a 3.5% annual increase until 2050, assuming ambitious efficiency improvements^5^.

It has been shown that forecasting electricity demand is a complex process given its reliance on different interdependent aspects including but not limited to economic, social, climatic and political factors^6–8^. In parallel, robust electricity demand projections play a central role in strategic planning for energy security, including decisions related to electricity imports and exports, cross-border interconnections, and regional power market integration, particularly in systems with increasing reliance on variable renewable energy sources^9,10^. Furthermore, reliable forecasting supports the effective deployment and operation of co-generation and distributed energy systems, improving overall system efficiency, and reducing fuel consumption and operational costs^11,12^. From a reliability perspective, accurate demand forecasts are essential for mitigating the risk of power shortages and large-scale power outages, as they inform adequacy assessments, maintenance scheduling, and contingency planning^13^. Such forecasts also underpin long-term policy alignment with climate objectives, including net-zero commitments, by balancing the integration of low-carbon technologies with conventional generation alternatives^14^. Subsequently, electricity demand forecasting research has traditionally employed both statistical and machine-learning approaches, broadly classified into univariate models that are based solely on historical demand and multivariate models that incorporate explanatory variables such as weather, economic activity, and population growth^15,16^.

*Univariate* electricity demand forecasting approaches have traditionally relied on Autoregressive Integrated Moving Average (ARIMA) and Seasonal ARIMA (SARIMA) models due to their transparency and ease of interpretation. One study employed an ARIMA model for medium- and long-term forecasting and found the monthly horizon to yield the most reliable results^17^, while comparisons between SARIMA and Artificial Neural Networks (ANNs) reported modest accuracy gains despite additional computational effort^18^. Hybrid and ensemble univariate models have further improved performance by capturing nonlinear residuals, including SARIMA–neural network hybrids^19^ and ensemble approaches combining deterministic and stochastic components^10^. More recently, deep learning methods, particularly Recurrent Neural Networks (RNNs) including Long Short-Term Memory (LSTM) and Gated Recurrent Units (GRUs), have been increasingly applied in univariate electricity forecasting due to their ability to capture temporal dependencies. LSTM-based approaches have consistently outperformed traditional statistical models across various regional contexts^20^, while RNN-based frameworks have demonstrated high predictive accuracy for household- and smart-meter-level consumption^21^. GRUs, a simplified gated variant of LSTM, have also been widely investigated, with comparative studies reporting performance comparable to LSTM models while offering reduced computational complexity and fewer parameters, making them computationally efficient alternatives for univariate electricity demand forecasting applications^22,23^. In parallel, traditional machine-learning algorithms, including Random Forests, K-Nearest Neighbors (KNN), Support Vector Machines (SVM), and Gradient Boosting, have also been widely applied, with mixed comparative performance depending on temporal resolution and application context^24,25^. Despite these advances, univariate models assume relatively stable long-term trends and remain limited in capturing the impacts of climate variability, economic shocks, demographic shifts, and policy changes on electricity consumption^26–28^, reducing their suitability for national-scale, long-term energy planning.

To address such model limitations, *multivariate* forecasting approaches have emerged through incorporating additional explanatory variables beyond historical demand. Short-term multivariate models often rely on high-resolution data, such as hourly or daily observations. Examples include LSTM-based frameworks integrating demand, weather, and socioeconomic indicators^29^, as well as large-scale smart-meter studies capturing detailed building and climate characteristics^30^. While effective for operational and localized forecasting, such approaches are less applicable to long-term, national-scale forecasting due to their reliance on high-frequency data. In contrast, long-term electricity demand forecasting typically employs lower-resolution datasets, such as monthly or annual records, to capture macro-level trends. Prior studies have incorporated limited combinations of economic, demographic, and climatic variables across diverse regions. Examples include models integrating GDP, population, and temperature scenarios in Ireland^31^; decomposition-based analyses in Hong Kong and Singapore considering GDP, population, and climate indices^32^; and regression-based approaches in Ontario using socioeconomic and weather indicators^33^. More recent studies have explored advanced machine-learning and deep-learning models, such as hybrid XGBoost–CatBoost frameworks^34^, stacked autoencoders^35^, and multivariate LSTM variants incorporating meteorological variables^36^. Scenario-based forecasting approaches combining regression and time-series models have also been proposed to assess long-term national demand under alternative development pathways^37^. While these studies demonstrate that multiple statistical, machine-learning, and deep-learning models can achieve competitive forecasting performance, the primary limitation emerging across literature lies less in model selection and more in the breadth and integration of the explanatory variables used for long-term forecasting.

To date, multivariate electricity demand forecasting studies continue to depend on a limited number of inputs, with most models depending primarily on historical demand data and a small subset of economic or climate-related variables. Such narrow feature scope limits the capacity of existing models to represent the complex drivers underlying long-term electricity consumption, which are central to national-level strategic planning. To bridge this gap, this study introduces a reproducible temporal three-phase forecasting system explicitly designed for long-term, national-level electricity consumption. The proposed approach emphasizes the structured integration of shallow and deep learning models with planning-oriented set of features to support stable and reliable forecasts over extended time spans. As such, a key characteristic of the developed system is its holistic incorporation of multi-domain predictors, encompassing energy generation, climatic, economic, and social dimensions, allowing the system model to capture interactions across domains that shape long-term demand trends. Specifically, the developed system is deployed within a standardized workflow that performs systematic feature integration through imputation and temporal upsampling, coupled with controlled hyperparameter optimization using Optuna to maximize the validation Coefficient of Determination (R²). This standardized workflow mitigates common limitations of prior studies, such as reliance on narrow or difficult-to-obtain indicators and enhances the robustness and practical relevance of long-term electricity forecasts for real-world national electricity planning. The developed system was deployed on Egypt, as a demonstration application to highlight the system utility. Finally, it is important to note that the developed three-phase system is designed to be transferable across national contexts, as it is grounded in methodological principles that are independent of country-specific demand characteristics. As such, the methodology addresses universal challenges inherent to long-term electricity consumption forecasting, including model architecture development, mitigation of overfitting, and modeling of non-linear feature interactions.

## Electricity consumption prediction system

**Fig. 1:**
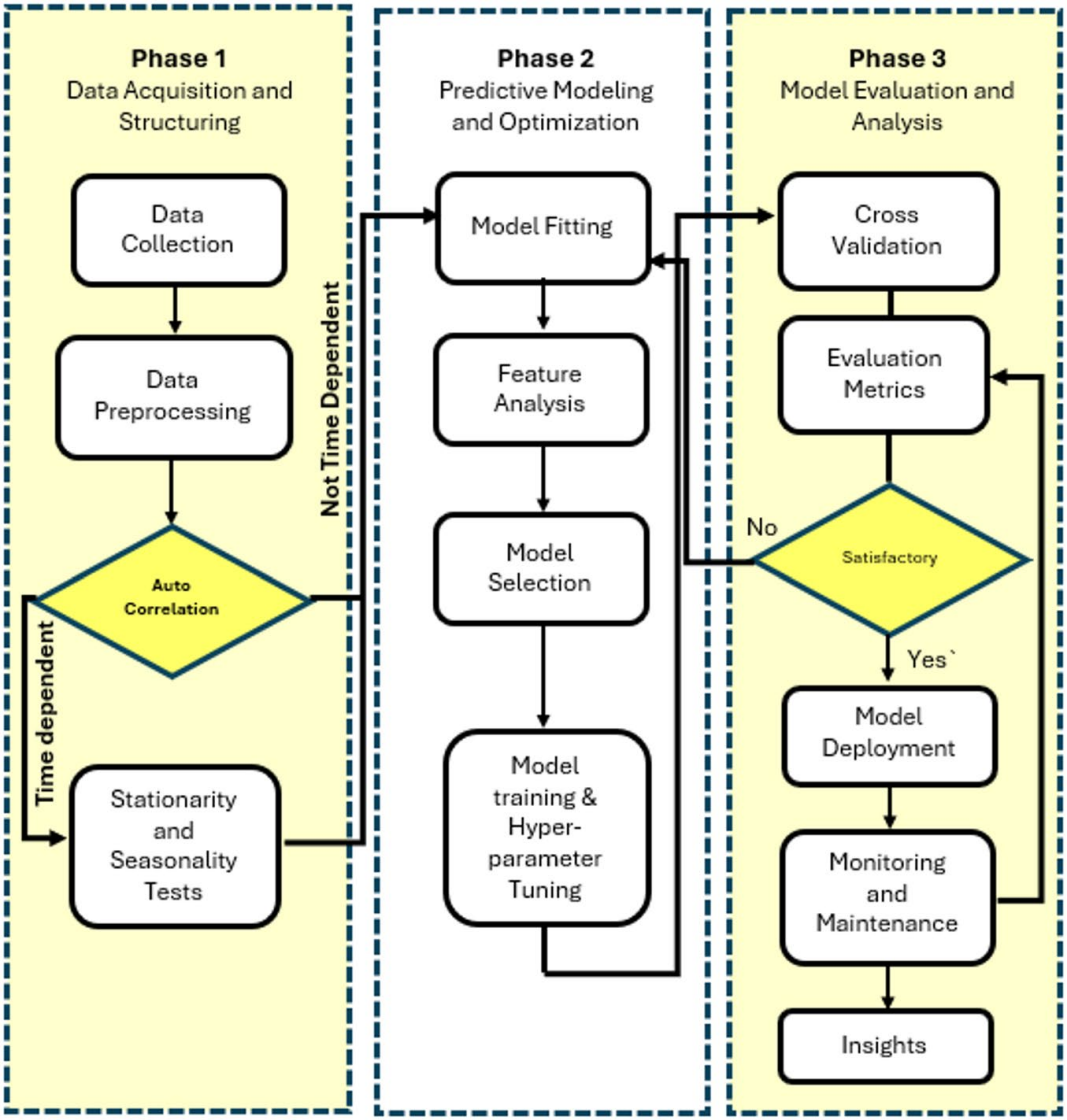
Three-phase system for electricity consumption forecasting.

Figure 1 presents the proposed structured three-phase system for developing a national-wide electricity consumption forecasting model which entails: *i*) Data Acquisition and Structuring, which covers the collection, preprocessing, and diagnostic assessment of relevant features across temporal and spatial scales; *ii*) Predictive Modeling and Optimization, where suitable machine learning algorithms are selected, configured, and trained on the prepared dataset; and *iii*) Model Evaluation and Analysis, where model performance is rigorously tested and the finalized model is deployed. This phased structure provides a transparent, scalable, and methodologically sound foundation for forecasting electricity demand.

### Phase 1: data acquisition and structuring

Phase 1 of the proposed system focuses on data collection and preprocessing, aiming to construct a feature set capable of representing the drivers of national electricity demand over long planning horizons. Selected variables are classified under the following four broad domains: (1) Electricity Indicators, (2) Climatic Indicators, (3) Economic Indicators, (4) Population Dynamics Indicators.

Electricity indicators include generation capacity to account for supply-side constraints, which is essential for long-term infrastructure planning^38^. Moreover, environmental conditions exert a strong influence on electricity demand through both seasonal and extreme events. Temperature variables—including average mean surface temperature, average maximum surface temperature, and maximum daily temperature—capture baseline conditions and extreme heat events that drive cooling demand. For example, in Dhaka, a 1 °C reduction in air temperature could save 81 MW of electricity, while sub-Saharan Africa records a 6.7% increase in demand for each 1 °C rise^39,40^. Relative humidity also interacts with temperature in shaping thermal comfort and energy use, while precipitation captures broader seasonal conditions that may indirectly affect demand^41,42^.

On the other hand, economic indicators play a significant role, albeit indirect, in explaining variations in electricity consumption. GDP per capita serves as a proxy for industrial production intensity, household purchasing power, and overall economic activity, and is found to account for approximately 14% of the total variation in electricity demand^43,44^. In addition, external debt explains nearly 10% of the observed consumption variability, underscoring the influence of macroeconomic stability on individual household energy consumption patterns^44,45^. Moreover, official reserve assets and Consumer Price Index (CPI) provide additional context on economic stability and purchasing power^46^, while unemployment rate represents labor market conditions that indirectly influence electricity demand through their effect on economic activity^47^. Furthermore, urbanization drives higher energy use, with a 1% increase in urban population associated with a 0.85–3.87% rise in electricity consumption across South Asian countries^48,49^ Additionally, energy consumption demonstrates distinct correlation patterns with urban and rural population growth, underscoring the need to incorporate both features while forecasting electricity demand^50^. Finally, tourism contributes to short-term seasonal spikes: in Malaysia, a 1% increase in tourist arrivals, expenditures, or receipts leads to long-term electricity consumption increases of 0.46–0.64%, and short-term increases of 0.30–0.51%^51^.

Given that data preprocessing is a critical preliminary stage in time series analysis, after data collection Phase 1 of the proposed system focuses on cleaning and integrating the collected data. This includes addressing inconsistencies, managing missing values, and harmonizing datasets with different temporal resolutions, so all variables align on a consistent time scale^52^. Various methods exist for data imputation, ranging from simple approaches such as forward/backward filling or linear interpolation to more advanced techniques such as Piecewise Cubic Hermite Interpolation (PCHIP). Simple methods are computationally efficient and can work for short gaps or stable variables, but they often fail to capture nonlinear trends, monotonic behavior, or seasonal patterns in dynamic time series data. To address these challenges, the proposed system employs a differentiated methodological approach, selecting the most appropriate imputation technique based on the distinct characteristics and behavioral patterns of each variable. As such, cubic spline interpolation fits smooth polynomial curves through data points, making it suitable for continuous variables with gradual nonlinear trends^10,53^. In contrast, PCHIP preserves the monotonicity and overall shape of variables by constructing piecewise cubic polynomials that respect the slope at each observed point, preventing overshooting and ensuring that interpolated values stay within the range defined by neighboring points. This makes it particularly suitable for steadily increasing or decreasing series^54,55^. Model-based approaches, such as ARIMA or SARIMA, leverage the statistical structure of the series to handle variables with autocorrelation, trend, or seasonality. SARIMA, in particular, is effective for cyclic or seasonal data, such as climate variables, but requires careful parameter tuning and is computationally more intensive than interpolation^56^.

Once preprocessing is complete, it is essential to evaluate the temporal characteristics of each variable, particularly time dependence and seasonality, as these directly inform the choice of subsequent modeling techniques. The first step is to construct a temporal plot, providing a preliminary visual assessment of trends or repeating seasonal patterns. To quantify these patterns, the series can be decomposed into trend, seasonal, and residual components, revealing structures not immediately apparent in raw data. Following decomposition, the Auto Correlation Function (ACF) and Partial Auto Correlation Function (PACF) plots are employed to assess the extent of time dependence and potential seasonality. The ACF plot illustrates the correlation between the time series and its lagged values across various lag intervals where significant autocorrelation at specific lags suggests the presence of seasonal cycles. In contrast, the PACF plot measures the correlation between the time series and its lagged values while controlling for the effects of shorter lags. This plot is instrumental in determining the number of autoregressive terms that may be necessary in models such as ARIMA. The final step in diagnosing the temporal behavior of the series involves conducting the Augmented Dickey-Fuller (ADF) test to evaluate stationarity. The ADF test estimates the following regression equation:

$$\:\varDelta\:{y}_{t}=\:\alpha\:+\:\beta\:t+\gamma\:{y}_{t-1}+\:\sum\:_{i=1}^{\rho\:}{\delta\:}_{i}\varDelta\:{y}_{t-1}+{ϵ}_{t}$$

where $\:\varDelta\:{y}_{t}$​ is the first difference of the series, $\:t\:$represents a deterministic trend, and $\:{ϵ}_{t}\:$is white noise. The test specifically evaluates the null hypothesis $\:{H}_{0}$: $\:\gamma\:$ = 0, which posits that the time series contains a unit root and is thus non-stationary, against the alternative hypothesis $\:{H}_{1}$: $\:\gamma\:$ < 0, which suggests that the series is stationary. A key output of the test is the p-value, which indicates the probability of observing the test statistic under the assumption that the null hypothesis is true. If the p-value exceeds 0.05, the null hypothesis cannot be rejected, implying that the series is non-stationary and may exhibit trends or seasonal effects. Conversely, a p-value less than or equal to 0.05 indicates stationarity, where the series maintains constant statistical properties over time.

The resulting patterns in the ACF and PACF plots, along with the differencing requirements indicated by the ADF test, provide a clear indication of temporal dependence in the dataset. Gradual decay in the ACF, significant spikes in the PACF at specific lags, or repeated seasonal peaks signal that past values influence current observations, demonstrating time dependence or seasonality. Conversely, series with weak or rapidly decaying autocorrelations and minimal differencing requirements exhibit little temporal structure, indicating that their values are largely time independent.

### Phase 2: predictive modeling and optimization

Phase 2 commences with model fitting and comprehensive feature analysis, serving as a foundation for effective predictive modeling. The goal of this phase is to investigate the structural characteristics of the dataset, identify patterns, and understand the relationships among variables to guide model selection and improve performance.

The initial step in this process involves the use of statistical filter methods to identify correlated features. Pearson’s correlation coefficient, a widely adopted linear filter method, quantifies the strength and direction of linear relationships between pairs of variables. While it offers computational simplicity and interpretability, Pearson’s correlation may fail to detect nonlinear associations. In such cases, Spearman’s rank correlation, a non-parametric technique, provides a more robust alternative^57^. Spearman’s method assesses the monotonic relationship between variables by evaluating the ranks rather than actual values, making it well-suited for datasets where variables are non-linearly dependent or not normally distributed.

Following feature analysis, model selection is guided by the temporal characteristics of the dataset—specifically, whether the data exhibits time dependence or seasonal behavior. For datasets without temporal structure, a range of classical machine learning models may be applied. Linear Regression assumes a direct, additive relationship between predictors and the response variable, making it suitable for interpretable, low-complexity applications^58^. In contrast, SVM is powerful in high-dimensional spaces and can model both linear and nonlinear relationships using kernel functions^59^. For more intricate datasets with complex patterns, ensemble models such as Random Forest, Gradient Boosting, XGBoost, and LightGBM are employed. These models are particularly effective at capturing non-linearities, handling missing data, and managing large feature sets, thereby providing high accuracy and resilience to overfitting^60^. In cases where the dataset exhibits time dependence or seasonality, time series models are more appropriate. ARIMA is a statistical model that combines autoregression (AR), differencing (I), and moving average (MA) components to model non-seasonal time series data with trend and autocorrelation. For data with seasonal patterns, SARIMAX extends the ARIMA model by incorporating exogenous features, seasonal differencing and seasonal AR and MA components, making them suitable for periodic fluctuations. These models rely on stationarity assumptions and are generally effective for univariate, moderately complex forecasting tasks^61^.

However, in scenarios involving multivariate time series, long-term dependencies, or non-stationary and nonlinear dynamics, deep learning approaches provide a more robust solution. LSTM and GRU networks are specialized types of RNNs designed to overcome the vanishing gradient problem associated with traditional RNNs^62,63^. They maintain memory over long sequences and can capture intricate temporal patterns in sequential data. These models are particularly advantageous in applications involving weather data, economic indicators, energy consumption, and other real-world time series where temporal dependencies are strong and complex^64^. Among RNN variants, GRUs provide several key advantages, including greater computational efficiency^65^. By combining the forget and input gates into a single update gate, GRUs simplify the network architecture, reducing the number of parameters and enabling faster training, while still achieving comparable or superior performance on many sequential prediction tasks^63,66^. Their efficiency, coupled with the ability to retain long-term dependencies, makes GRUs particularly well-suited for datasets with long sequences or limited training data^67^, making them a practical and effective choice for large-scale, real-world forecasting applications. Moreover, GRU networks, while underrepresented in electricity forecasting, have demonstrated higher accuracy and lower error rates than LSTM and vanilla RNNs when applied to long-term prediction tasks, such as temperature forecasting^68^, solar power generation^69^ and electricity load prediction^70^.

To enhance model performance and ensure optimal architecture selection, the framework employs Optuna, a hyperparameter optimization framework, to systematically search for the best network configurations. After hyperparameter optimization, SHAP-based interpretability (SHapley Additive exPlanations) is employed to evaluate feature contributions. This approach is selected because it provides a model-agnostic, performance-driven measure of feature relevance, making it particularly suitable for nonlinear deep learning models and long-term forecasting^71^. The least contributing features are iteratively removed, and the model’s performance is tracked over multiple random weight initializations to ensure robustness and stability of the predictions.

### Phase 3: model evaluation and analysis

Phase 3 focuses on evaluating the performance of the developed models based on their accuracy, consistency, and robustness. In this stage, the dataset is typically divided into three sets: a training set, a validation set, and a testing set. The training set is used to train the models, the validation set helps tune model parameters, and the testing set is reserved for final evaluation. It is crucial that this split occurs before any scaling or normalization to avoid data leakage, ensuring that information from the validation or testing sets does not influence the training process. This provides a proper estimate of the models’ generalization ability.

To enhance the reliability and stability of model evaluation, cross-validation techniques are applied. For traditional regression models, tree-based learners, or ensemble methods such as Random Forest and XGBoost, K-Fold Cross-Validation is often used to average performance across multiple data splits, reducing sensitivity to data variance and overfitting. However, for time series forecasting, particularly with RNNs such as LSTM and GRU, standard cross-validation is inappropriate due to the sequential nature of the data. Instead, a chronologically split validation set, typically comprising 5–10% of the data, is used to monitor performance during fine-tuning the model. This preserves temporal order and prevents data leakage, which is critical for time-dependent tasks.

To evaluate model performance, several metrics are used to assess predictive accuracy and error characteristics. The R² quantifies how well predictions match observed values, with higher values indicating better fit. The Mean Absolute Error (MAE) measures the average absolute difference between predicted and actual values, while the Root Mean Squared Error (RMSE), sensitive to large errors, provides an intuitive measure in the same unit as the target variable. In this study, RMSE is expressed as a percentage of the mean of testing data to contextualize error relative to typical consumption levels. If performance is satisfactory, the model is deployed. Otherwise, it loops back to Phase 2 for further tuning or algorithm reselection.

## System demonstration application: electricity consumption forecasting in Egypt

### Phase 1: data acquisition and structuring

The proposed framework was implemented to forecast national-scale electricity consumption in Egypt, a country experiencing rapid urbanization and sustained growth in electricity demand. Egypt presents a particularly relevant case study due to the combination of climate-driven demand surges and emerging supply-side constraints. Over the past couple of decades, electricity demand had increased substantially alongside socioeconomic development, with peak loads rising sharply and are still projected to grow at high annual rates^72^. Recent heat waves further intensified electricity demand, pushing national loads to record levels and highlighting the strong dependence of consumption on extreme temperature events^73^. Despite the presence of nominal reserve capacity, Egypt experienced recurring power rationing since 2023, driven in part by fuel supply limitations and macroeconomic pressures rather than demand growth alone^74^. Declining domestic natural gas production and increasing reliance on imports introduced additional constraints on electricity generation, linking electricity consumption patterns to broader economic indicators such as external debt and reserve assets^75^. These interacting climatic, social and economic factors underscore the complexity and significance of long-term electricity demand forecasting in Egypt.

The first phase of the proposed system therefore focused on data preparation, involving the collection of variables from reliable and authoritative sources, their organization into meaningful domains, and the alignment of all features to a consistent temporal resolution. The selection and justification of features are established in Phase 1 of the framework (i.e., Sect. 2.1) according to their relevance to electricity demand dynamics, and Table 1 provides a summary of the selected feature set which spans from January 2000 to December 2023. In this study, climate data were sourced from the World Bank Climate Change Knowledge Portal^76^, while the rest of the features were obtained from the CEIC portal^77^.

**Table 1:** List of Input Features for Energy Consumption Prediction.

Denotation	Feature Name	Domain	Period	Units	Frequency
Y1	Electricity Consumption	Electricity Indicators	Jan-00 to Dec-23	KWh mn	Monthly
X1	Electricity Generation Capacity		Jun-00 to Jun-23	MW	Annually
X2	Precipitation	Climatic	Jan-00 to Dec-22	mm	Monthly
X3	Average Mean Surface Temperature		Jan-00 to Dec-22	°C	Monthly
X4	Average Maximum Surface Temperature	Indicators	Jan-00 to Dec-22	°C	Monthly
X5	Relative Humidity		Jan-00 to Dec-22	%	Monthly
X6	Maximum Daily Temperature		Jan-00 to Dec-22	°C	Monthly
X7	GDP per Capita	Economic Indicators	Jun-00 to Jun-23	USD	Annually
X8	External Debt		Mar-00 to Dec-23	USD mn	Quarterly
X9	Official Reserve Assets		Dec-04 to Dec-23	USD mn	Monthly
X10	Consumer Price Index (CPI) Growth Rate		Jan-00 to Dec-23	% (YoY)	Monthly
X11	Unemployment Rate		Mar-03 to Dec-23	%	Quarterly
X12	Urban Population	Population Dynamics Indicators	Jun-00 to Jun-23	Person	Annually
X13	Rural Population		Jun-00 to Jun-23	Person	Annually
X14	Tourist Influx		Jan-00 to Jun-22	Person	Monthly

**Fig. 2:**
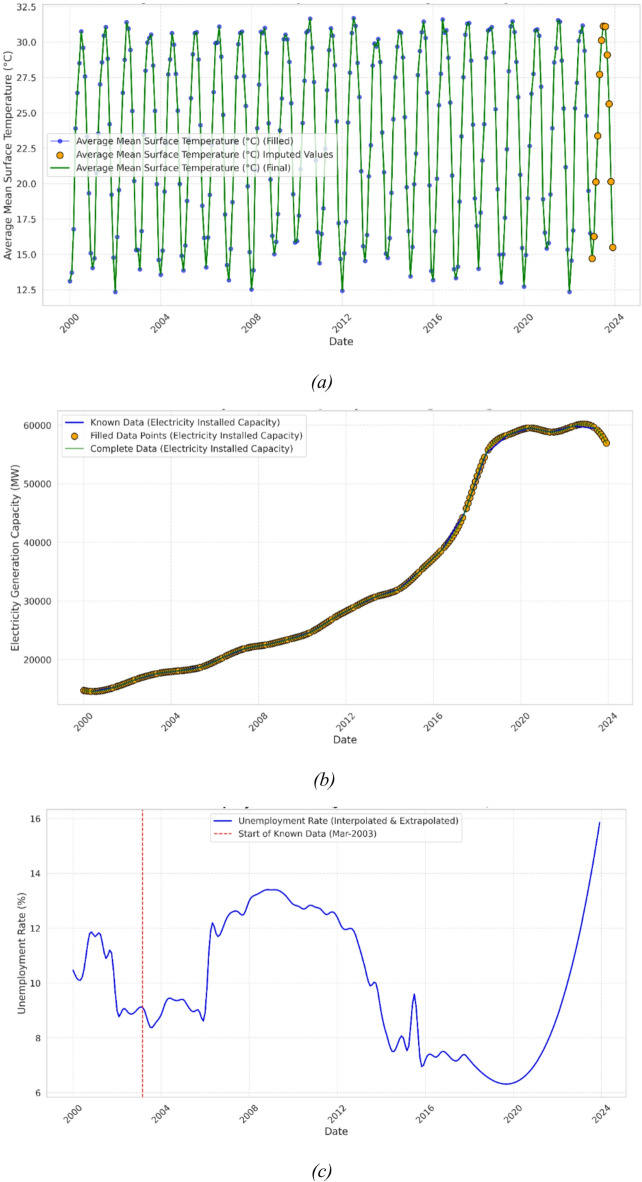
- Data Interpolation Techniques (**a**) Time Series Interpolation; (**b**) Cubic Spline Interpolation; (**c**) Piecewise Cubic Hermite Interpolation.

To address the discrepancy between the time frames and frequencies of the collected variables, upsampling techniques which were discussed in Sect.  2.1 were utilized to transform the entire dataset into monthly data. As such, each variable was first decomposed into its seasonality, trend, and residual components to identify its underlying patterns, which guided the selection of the most suitable upsampling technique. Features exhibiting seasonal trends, such as climatic indicators and tourist influx were upsampled using SARIMA, as shown in Fig. 2(a). A uniform order of (, , ) = (1, 1, 1) and (,,,) = (1,1,1,12) was applied across all seasonal features. The seasonal period s = 12 reflects the intrinsic annual cycle, while the ARIMA components (1,1,1) provide a simple, consistent model for short-term dependencies and trends. SARIMA was particularly effective in capturing the periodic nature of these variables, ensuring that the extended data maintained logical and realistic seasonal patterns. Alternatively, cubic spline interpolation was applied to features characterized by steady upward trends, such as ‘External Debt’, Electricity Installed Capacity’, Urban Population’, Rural Population’, and ‘GDP per capita’, as illustrated in Fig. 2(b). This method provided smooth and continuous transitions between data points while preserving the consistent growth trends inherent in these features. Finally, monotonic features such as the ‘Unemployment Rate’, which consistently increased or decreased over time, were up sampled using PCHIP, as shown in Fig. 2(c), for its ability to preserve monotonicity and prevent overshooting.

**Fig. 3:**
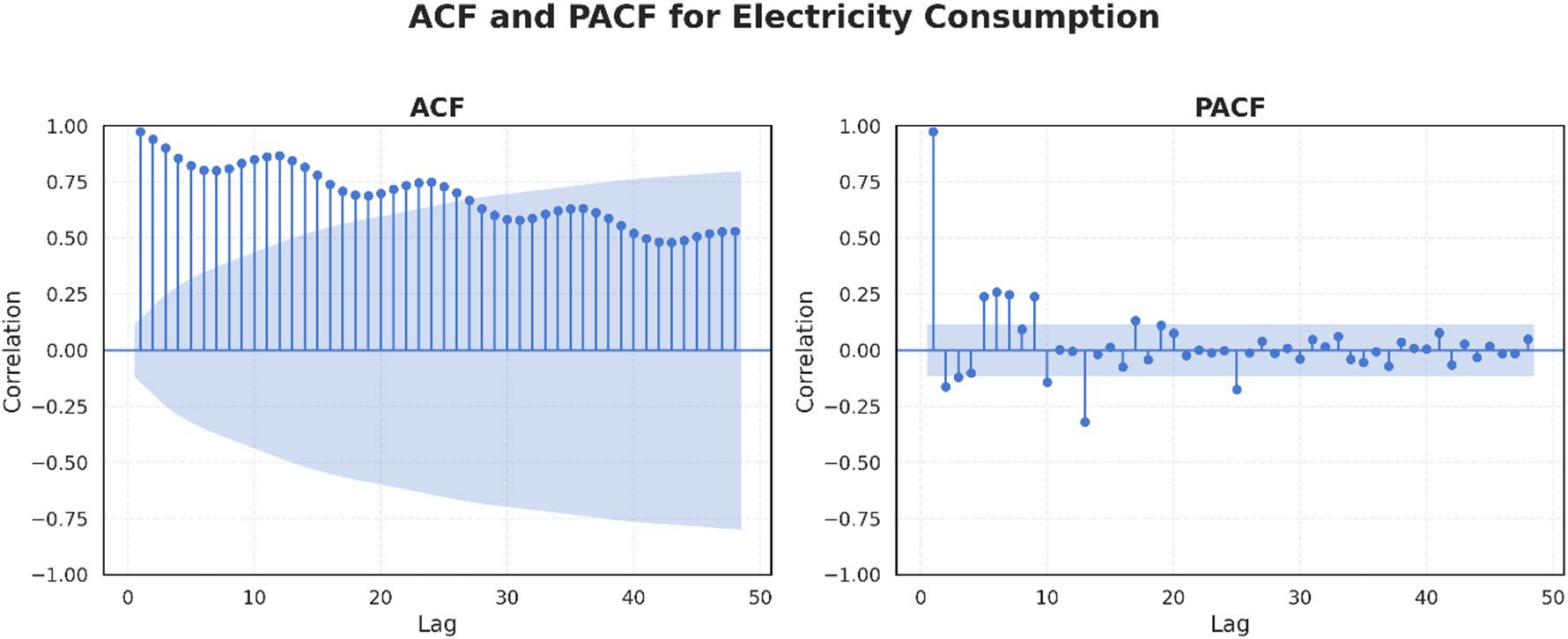
– Electricity Consumption ACF and PACF.

The next analytical step involved examining the temporal dependencies within the dataset through the ACF and PACF plots. These plots illustrate the degree to which current observations are correlated with their historical values, providing insight into both short- and long-term dependencies. To capture a comprehensive temporal structure, 48 lags were selected—equivalent to four years of monthly data—allowing for the detection of seasonal cycles and longer memory effects. Figure 3 presents the ACF and PACF results for the target variable, Electricity Consumption, with a 95% confidence interval, where spikes extending beyond the threshold are deemed statistically significant. The ACF plot exhibited a gradual decay with prominent spikes at regular 12-month intervals, revealing strong annual seasonality in the monthly data. Meanwhile, the PACF plot showed a sharp, significant spike at lag 1, indicating a strong dependence on the value of the previous month. Additionally, lags 5, 6, 7, and 9 display moderate but significant partial autocorrelations, suggesting mid-range temporal dependencies. Notably, lags 13 and 25 show significant negative partial autocorrelations (approximately − 0.3 and − 0.2, respectively), which occur just after the one-year and two-year marks. These negative values implied an inverse seasonal relationship, meaning that if electricity consumption was high 13 or 25 months ago, it is more likely to be lower now, after controlling for the effects of the intermediate lags. Complementing these plots, the ADF test was employed to assess the stationarity of each time series. Differencing was applied iteratively until stationarity was achieved, with the number of differencing operations serving as an indicator of the variable’s complexity and dynamics. Table 2 presents a synthesis of the results from the ACF, PACF, and ADF analyses, including the number of differencing steps applied to each variable to attain stationarity.

The comprehensive temporal analysis summarized in Table 2 demonstrates pronounced seasonality, significant autocorrelations at multiple lags, and varying degrees of non-stationarity across the target variable and explanatory features. These characteristics strongly support the use of classical time series models such as ARIMA and SARIMA, which explicitly account for linear temporal dependencies and seasonal patterns after differencing to achieve stationarity. Furthermore, many features exhibit nonlinear and long-range dependencies indicated by oscillating autocorrelations and significant partial autocorrelations at mid- and long-term lags, which conventional linear models may not fully capture. Therefore, if ARIMA and SARIMA models fail to adequately represent these complex patterns, advanced sequence models such as GRU and LSTM RNNs can be deployed to leverage their capacity for modeling nonlinearities, memory effects, and variable-length temporal dependencies.

**Table 2:** Summary of Temporal Dependency Patterns.

Variable	ACF Pattern	PACF (Significant Lags)	Differencing Steps
Electricity Consumption	Gradual Decay with annual peaks	Several significant lags, notably at lags 13 and 25	1
Electricity Generation Capacity	Gradual decay without seasonal peaks	Lag 1 only	3
Precipitation	Strong seasonal autocorrelation (12-month cycle)	Several significant lags	0
Average Mean Surface Temperature	Strong seasonal autocorrelation (12-month cycle)	Several significant lags	0
Average Maximum Surface Temperature	Strong seasonal autocorrelation (12-month cycle)	Several significant lags	0
Relative Humidity	Strong seasonal autocorrelation (12-month cycle)	Several significant lags	0
Maximum Daily Temperature	Strong seasonal autocorrelation (12-month cycle)	Several significant lags	0
GDP per Capita	Gradual decay without seasonal peaks	Lag 1 only	3
External Debt	Gradual decay without seasonal peaks	Lag 1 only	2
Official Reserve Assets	Gradual decay; becomes negative at lag 25	Lag 1 only	1
Consumer Price Index (CPI)	Oscillatory decay; turns negative at lag 23 and trends back toward zero	Lag 1 and 13	1
Unemployment Rate	Gradual decay	Lag 1 and 2	2
Urban Population	Gradual decay	Lag 1	1
Rural Population	Gradual decay	Lag 1	2
Tourist Influx	Gradual Decay with annual peaks	Lag 1,3,12, 13 and 23	1

### Phase 2: predictive modeling and optimization

**Fig. 4:**
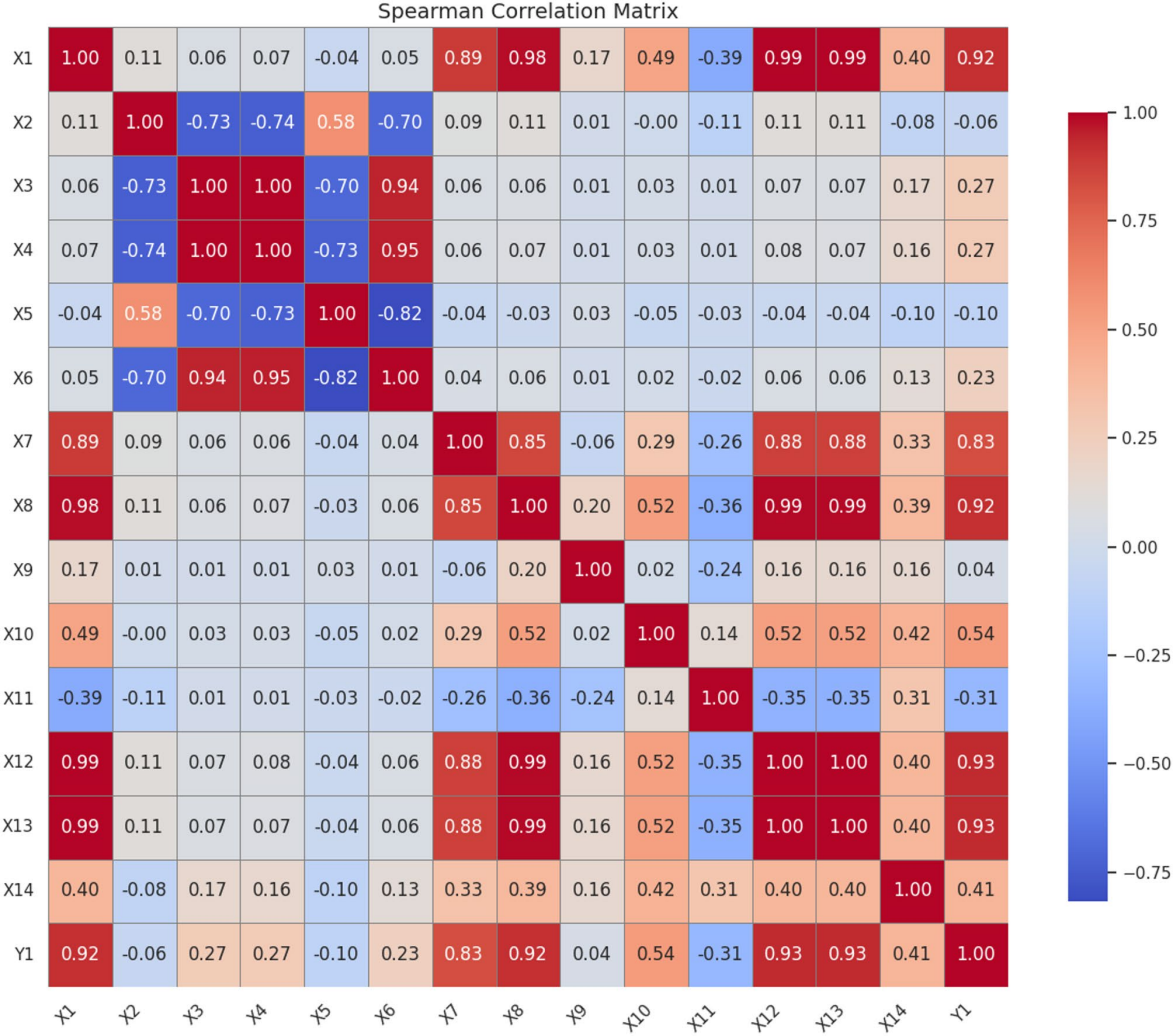
Spearman’s correlation for electricity consumption predictors.

In Phase 2, Spearman’s correlation was used to evaluate the strength and direction of monotonic relationships between electricity consumption and key input variables as shown in Fig. 4. Strong positive correlations can be observed between electricity consumption (Y1) and Electricity Generation Capacity ‘X1’ (ρ = 0.92), Urban Population ‘X12’ (ρ = 0.93), and External Debt ‘X8’ (ρ = 0.92), highlighting the impact of infrastructure expansion and urbanization on energy demand in Egypt. GDP per Capita ‘X7’ (ρ = 0.83) also displayed a strong positive association, reinforcing the link between economic prosperity and increased electricity usage.

Moderate correlations were observed with CPI ‘X10’ (ρ = 0.54) and Maximum Daily Temperature ‘X6’ (ρ = 0.23), suggesting that economic inflation and climatic extremes modestly influence consumption. In contrast, Precipitation ‘X2’ (ρ=−0.09) and Relative Humidity ‘X5’ (ρ=−0.10) showed weak correlations, indicating minimal long-term influence from weather patterns. Features such as Official Reserve Assets ‘X9’ (ρ = 0.04) and Unemployment Rate ‘X11’ (ρ=−0.31) exhibited limited relevance to electricity usage, supporting their exclusion from later feature selection stages. Overall, these findings affirmed the dominant role of socio-economic variables in shaping electricity consumption trends, and they validated the theoretical assumptions guiding this study.

The modelling phase began with the implementation of two classical time-series models—ARIMA and SARIMAX—as baseline benchmarks. The ARIMA model, a univariate approach, was trained on 90% of the log-transformed electricity consumption series, with candidate orders (p, d,q) evaluated using the Akaike Information Criterion (AIC) on the training subset. The model with the lowest AIC (*p* = 5, d = 1, q = 4) was selected and applied to the remaining 10% hold-out test set to generate out-of-sample forecasts, which were subsequently inverse-transformed for evaluation. To account for seasonal effects, a SARIMAX model was also trained on 90% of the dataset, incorporating non-seasonal parameters (p, d,q) = (1,0,0) and seasonal parameters (P, D,Q, m) = (1,0,1,12). Candidate non-seasonal and seasonal orders were systematically evaluated based on AIC on the training subset. The trained SARIMAX model was then used on 10% test subset to generate forecasts, which were inverse transformed to the original scale for evaluation. These classical models served as transparent, rigorously evaluated baselines for comparison with deep-learning alternatives, with all parameter selection performed solely on the training data to ensure the integrity of out-of-sample assessment.

**Fig. 5:**
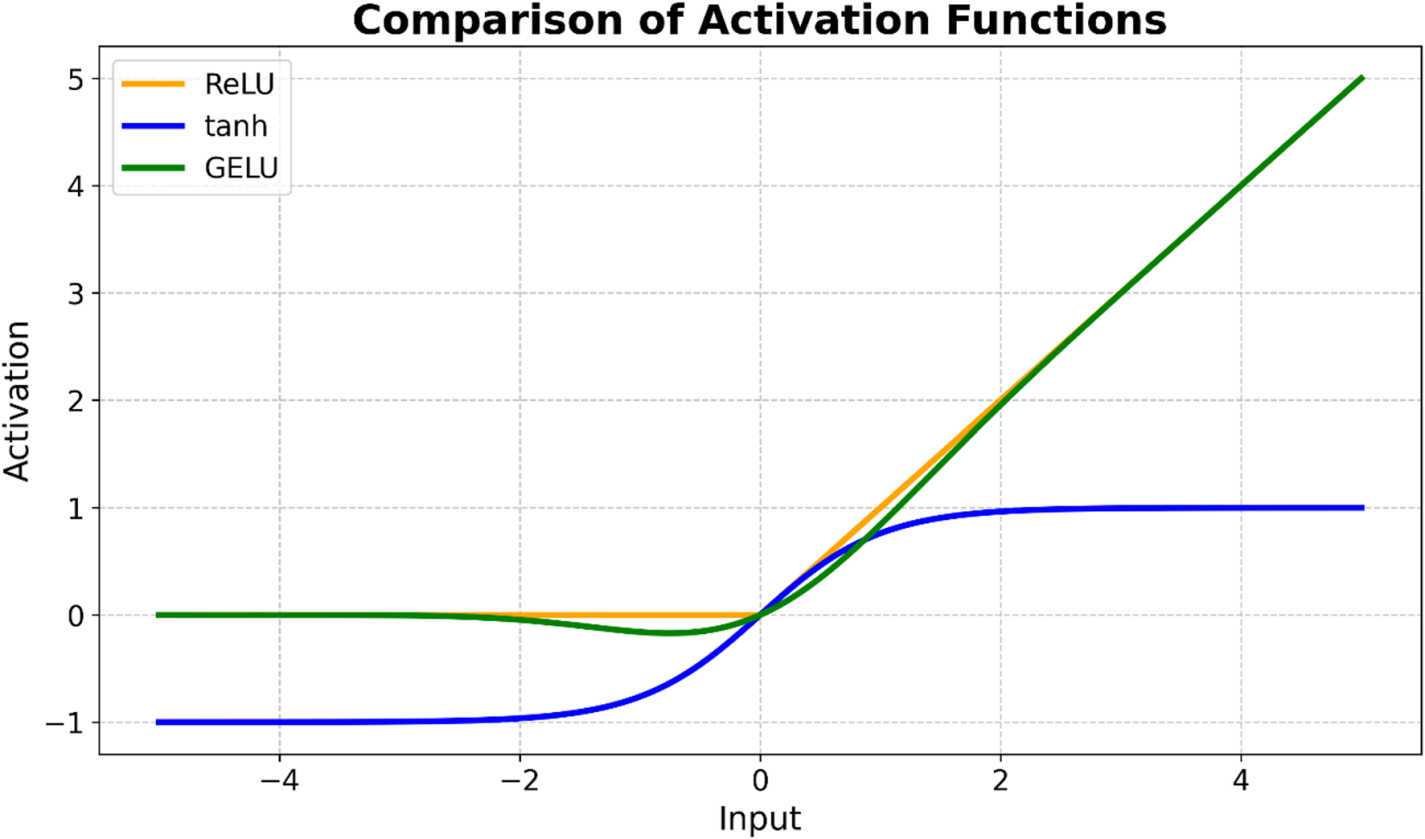
Comparison of activation functions.

GRU networks were employed to model multivariate electricity consumption, capturing both nonlinear relationships and long-term temporal dependencies. The dataset was temporally split into 85% for training, 5% for validation, and 10% for testing prior to normalization, after which Min–Max scaling was applied using statistics computed exclusively from the training set to prevent data leakage. All exogenous variables were restricted to information available up to time step , ensuring that no future information was introduced into the model inputs. GRU architectures with one, two, and three recurrent layers were constructed, and their hyperparameters were optimized using the automated Optuna framework by maximizing the validation R² score. Following hyperparameter selection, the best-performing configuration identified by Optuna for each architecture was subsequently evaluated across 20 independent runs with different random weight initializations. This post-optimization evaluation was conducted to assess the robustness of the models and to verify that the reported performance was not driven by favorable random initialization or overfitting to a particular training instance. All models employed the Gaussian Error Linear Unit (GELU) activation function, chosen for its smooth, probabilistic gating behavior that preserves small negative activations while mitigating extreme outputs, thereby improving generalization on noisy electricity consumption data. GELU was compared with conventional ReLU and tanh activations, showing superior performance in capturing both small fluctuations and larger trends as shown in Fig. 5.

Models also utilized the Adam optimizer with the mean squared error loss function and early stopping with a patience of 20 epochs, ensuring convergence while preventing overfitting. Weights were initialized using the Glorot (Xavier) normal distribution, which equalizes the variance of layer inputs and outputs, mitigating signal amplification or attenuation as depth increases and facilitating stable convergence. Recurrent weights were initialized orthogonally, ensuring that the spectral norm of the recurrent transformation remains close to unity, thereby preserving gradient magnitudes across time steps and preventing vanishing or exploding gradients during backpropagation through time. Additionally, gradient clipping (*clipnorm = 1*) was applied to further control extreme weight updates and prevent overfitting, which is particularly important given the limited size of the dataset.

**Fig. 6:**
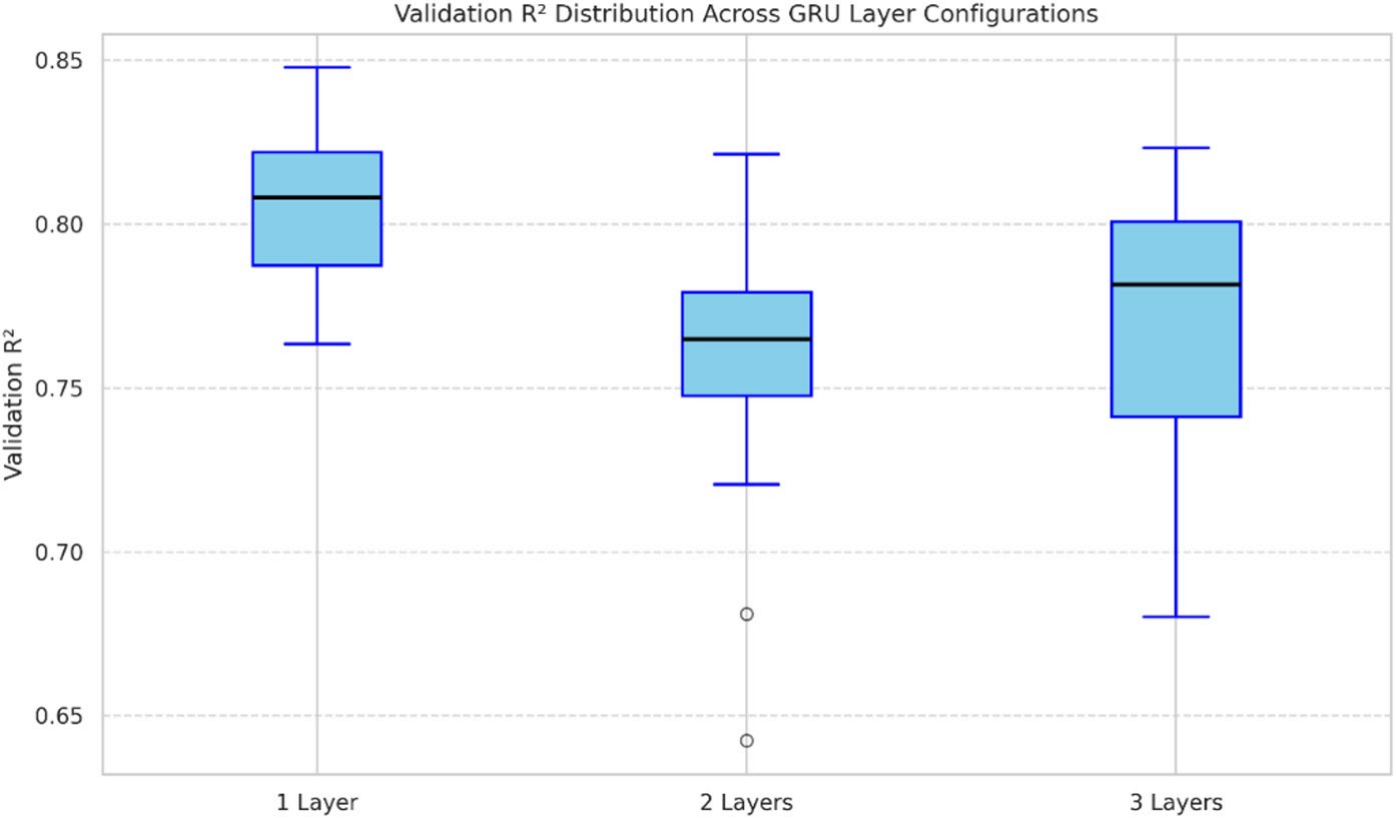
Boxplots of validation r² across 20 random weight initializations.

For the single-layer GRU, the optimal configuration consisted of 18 timesteps, 160 units, a dropout rate of 29.5%, a learning rate of 0.000286, and a batch size of 8. The two-layer architecture achieved optimal performance with 18 timesteps, 352 and 128 units in the first and second layers, dropout rates of 25.0% and 16.0%, a learning rate of 0.000533, and a batch size of 8. Finally, the three-layer GRU model was optimized with 22 timesteps, 128, 224, and 192 units in the first, second, and third layers, dropout rates of 11.47%, 22.67%, and 36.69%, a learning rate of 0.000317, and a batch size of 4. Among these configurations, the single-layer GRU achieved the most robust performance, attaining a mean validation R² of 80.55%, while the two- and three-layer models exhibited slightly lower mean validation scores of 75.75% and 73.19%, respectively. The deeper architectures showed greater variance across runs, achieving high R² in some cases but overfitting in others, reflecting the challenges of learning complex representations from a relatively small dataset. Figure 6 presents box plots summarizing the distribution of validation R² across all runs, highlighting both the stability of the single-layer network and the variability introduced by deeper configurations.

**Fig. 7:**
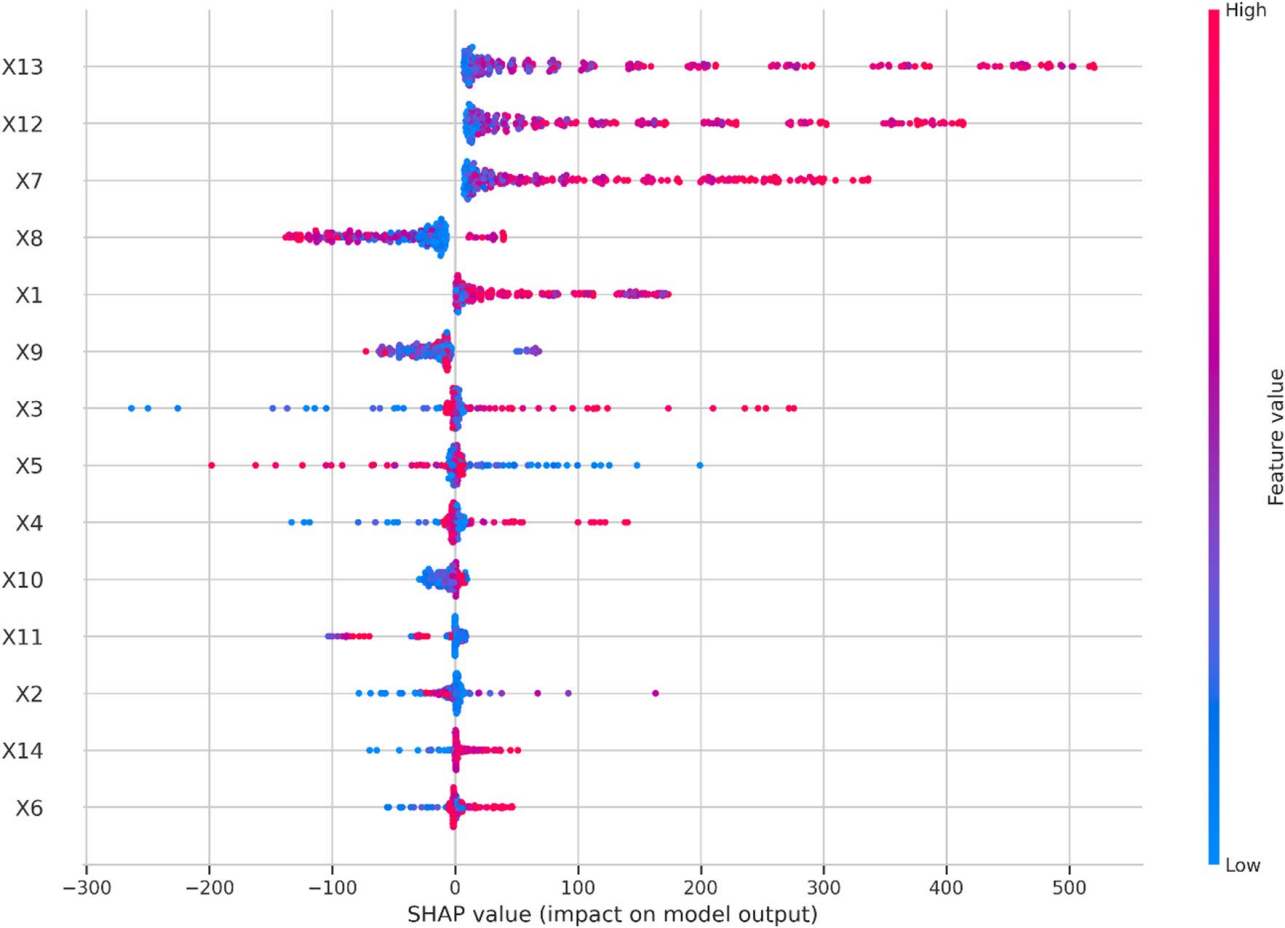
SHAP-based interpretability.

To further interpret the GRU’s predictions, SHAP-based interpretability was applied to the best-performing run, ensuring that input features accurately reflect the model’s predictive behavior. SHAP values were computed to quantify the influence of each feature value on model predictions showing that the top contributing features were Population Rural (X13), Population Urban (X12), and GDP/Capita (X7), while features such as Tourist Influx (X14) and Maximum Daily Temperature (X6) had minimal influence as shown in Fig. 7. A feature selection procedure was then conducted, where the least important features were removed iteratively, and the model was re-evaluated over five runs per subset to account for variability due to random weight initialization.

### Phase 3: model evaluation and analysis

Phase 3 focused on evaluating the developed electricity consumption prediction models in terms of accuracy, robustness, and suitability for forecasting. The ARIMA model achieved an R² score of 0.6159 and MAE of 624.25, with the RMSE representing 6.01% of the mean. In comparison, the SARIMAX model, which incorporates seasonal and exogenous components, yielded an R² of 0.3982 and MAE of 888.95, with the RMSE corresponding to 7.40% of the mean. While SARIMAX accounts for seasonality, it underperformed relative to ARIMA, indicating that the seasonal components alone were insufficient to capture electricity consumption patterns. The GRU model, consisting of a single recurrent layer, was selected due to its more stable performance compared with deeper architectures, as discussed previously. Evaluated across 20 independent runs with different random weight initializations, the single-layer GRU achieved a mean R² of 0.7989 and MAE of 518.69, with the RMSE corresponding to 4.42% of the mean demonstrating robustness and consistent performance.

**Fig. 8:**
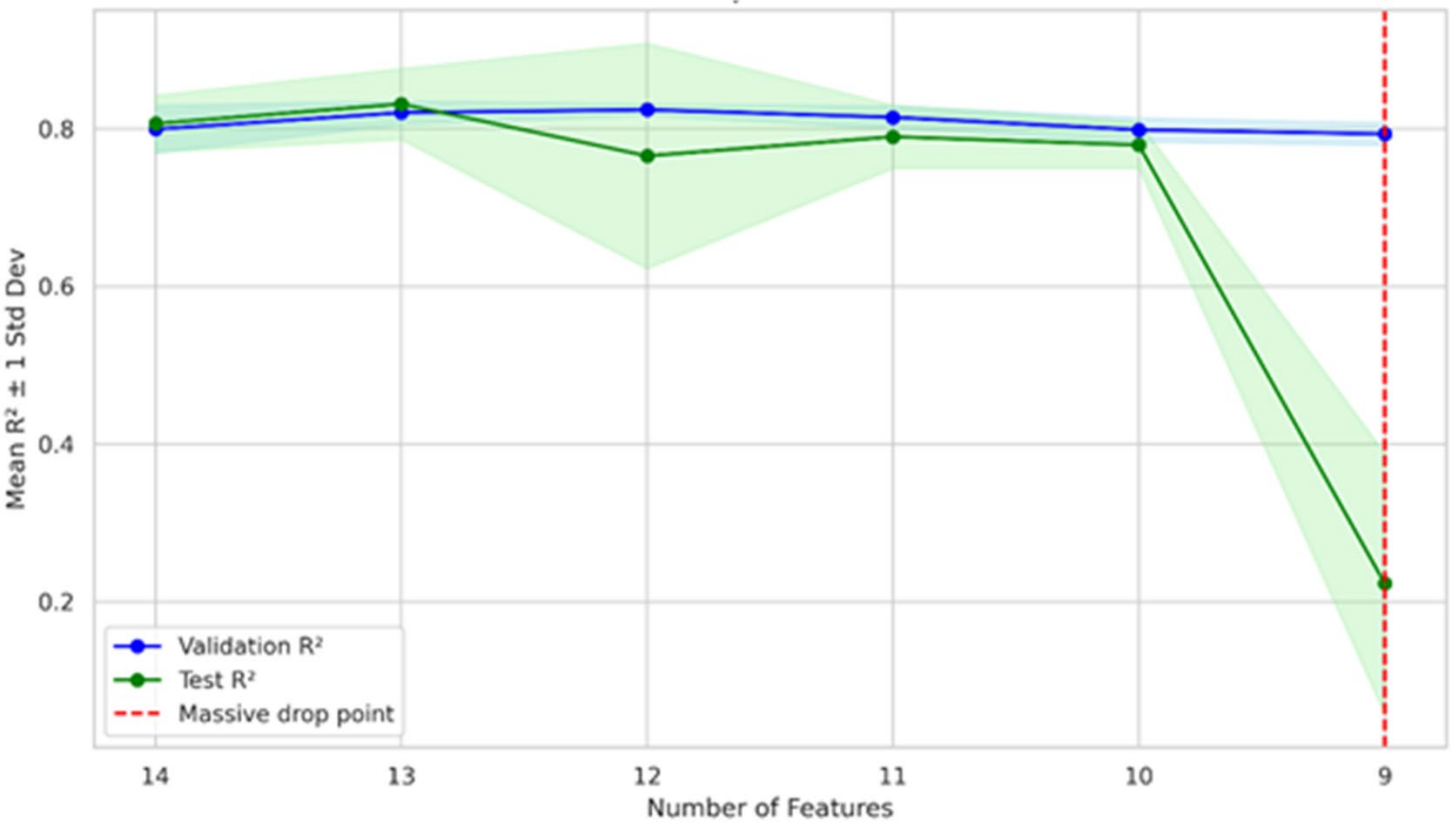
Impact of Feature Removal on R² Score.

Building on the single-layer GRU model, a systematic feature selection analysis, using the SHAP-based interpretability, was conducted to assess the trade-off between model complexity and predictive performance. Retaining 13 input features resulted in the strongest and most stable performance as shown in Fig. 8, achieving a mean R² of 0.8315, the lowest MAE of 489.02, and an RMSE equivalent to 4.05% of the mean. Reducing the feature set to 12 variables introduced occasional performance instability across runs, while further reductions to fewer than 10 features led to a substantial decline in R², indicating a loss of forecasting capability. Accordingly, the final model retained the most influential socio-economic, environmental, and electricity system variables, ensuring a robust balance between predictive accuracy and feature relevance.

**Fig. 9:**
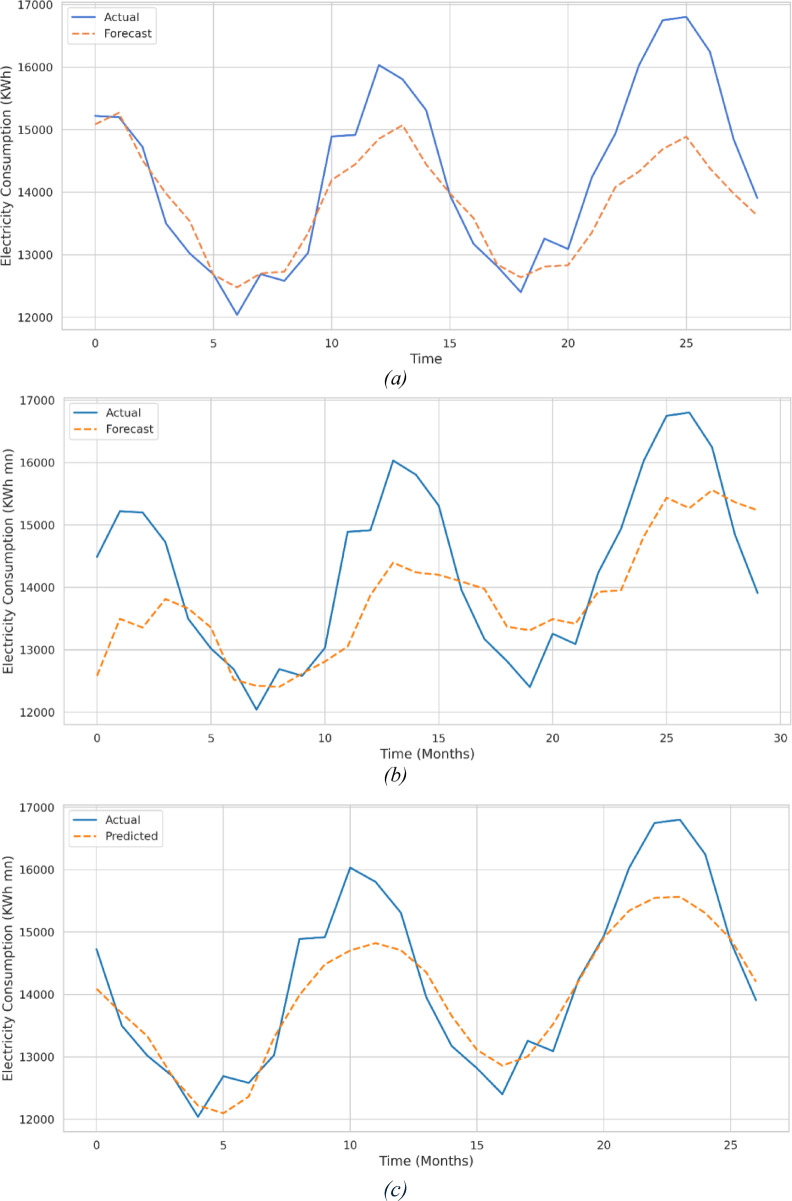

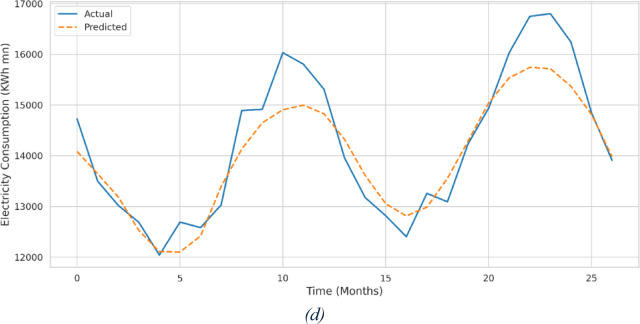
- Actual vs. Predicted Energy Consumption: (**a**) ARIMA, SARIMA, (**c**) GRU (All Features) and (**d**) GRU (Selected Features).

The results in Table 3 underscore the effectiveness of the tuned GRU model with targeted feature selection. Compared with ARIMA and SARIMAX, the GRU model generalized better to unseen data, capturing complex short- and long-term temporal dependencies while focusing on the most significant predictors through SHAP-based interpretability and feature selection. Figures 9(a)–(d) illustrate the actual versus predicted electricity consumption values for the testing dataset, showing that the GRU predictions aligned closely with observed consumption and outperformed both classical models. The sequence-based GRU architecture captured temporal patterns that ARIMA and SARIMAX models could not fully exploit, resulting in a more accurate and generalizable forecasting model.

**Table 3:** – Electricity consumption model evaluation metrics.

Model	*R* ^2^			MAE	Mean-RMSE
	Training	Validation	Testing		
ARIMA	0.971	-	0.615	624.25	6.01%
SARIMAX	0.986	-	0.398	888.95	7.04%
GRU (All Features)	0.974	0.806	0.799	518.69	4.42%
GRU (Selected Features)	0.977	0.831	0.832	482.09	4.05%

## Conclusions

This study presents a reproducible three-phase forecasting system for long-term, national-scale electricity demand. This work addresses a key gap in prior multivariate electricity demand forecasting, which often relied on limited feature sets confined to a single domain or restricted to historical demand and a few economic indicators. By systematically integrating economic, demographic, climatic, and energy system variables, and leveraging a time-series modeling framework with optimization-based hyperparameter tuning and feature selection, the system achieved a mean R² of 83.2% across multiple random weight initializations and a. mean RMSE equivalent to 4.05%, demonstrating both accuracy and robustness. The study further generated actionable insights through SHAP-based interpretability, identifying rural and urban population, GDP per capita and external debt as the most influential predictors. Increases in rural and urban population and GDP per capita consistently contributed to higher predicted electricity consumption, reflecting demographic growth and economic development, while external debt and generation capacity reflected historical patterns of incremental demand growth.

The proposed system provides a structured and reproducible tool for long-term electricity demand modeling, designed to support strategic energy planning and investment decisions. It enables stakeholders to perform scenario-based forecasting, exploring “what-if” conditions for generation capacity expansion, infrastructure investment allocation, and policy evaluation. By incorporating widely available and policy-relevant predictors, the framework allows stakeholders to project demand under different assumptions, ensuring that forecasts reflect the most influential drivers of electricity consumption. Moreover, the study also contributes to the literature by showing how multi-domain predictors and structured modeling workflows can improve interpretability and capture complex nonlinear dependencies, while SHAP-based interpretability provides actionable insights into the key drivers of demand. From a practical standpoint, the framework equips stakeholders, including policymakers, energy planners, and infrastructure developers, with a decision-enabling tool for scenario analysis, strategic energy investments, and capacity planning.

Future work could build on the system developed in this study in several ways. While the current implementation focused on development and validation, incorporating uncertainty quantification would be valuable for risk-informed decision-making. Scenario-based forecasting that explicitly captures potential variability in climate, economic, and policy conditions could further enhance the framework’s practical relevance. Additionally, the choice of input features in this study was guided by their wide availability and reliability across different countries, ensuring that the forecasting framework remains transferable and reproducible. While incorporating additional variables—such as electricity pricing, sectoral demand breakdowns, technology adoption trends, or efficiency and electrification policies—could further improve accuracy and tailor forecasts to specific national contexts, the selected economic and electricity indicators capture the main structural drivers of demand that reflect the broader challenges countries face in energy planning. Including more detailed or country-specific features could be considered in future applications to account for policy interventions, market dynamics, and long-term transition scenarios.

## Data Availability

The data used in this study are publicly available:1. Electricity, economic, population, and tourism indicators were obtained from the CEIC Data platform ( [https://www.ceicdata.com/en/country/egypt] )0.2. Climate-related variables were obtained from the World Bank Climate Knowledge Portal ( [https://climateknowledgeportal.worldbank.org/]).
